# Culturally Relevant Africultural Coping Moderates the Association Between Discrimination and Antiretroviral Adherence Among Sexual Minority Black Americans Living with HIV

**DOI:** 10.1007/s10461-023-04233-7

**Published:** 2023-12-07

**Authors:** Glenn J. Wagner, Laura M. Bogart, David J. Klein, Sean J. Lawrence, Kathy Goggin, Mahlet Gizaw, Matt G. Mutchler

**Affiliations:** 1 RAND Corporation, 1776 Main Street, P.O. Box 2138, Santa Monica, CA 90407-2138 USA; 2 APLA Health & Wellness, Los Angeles, CA USA; 3 Children’s Mercy Kansas City and University of Missouri - Kansas City Schools of Medicine and Pharmacy, Kansas City, MO USA; 4 California State University Dominguez Hills, Carson, CA USA

**Keywords:** Africultural coping, Discrimination, Adherence, Sexual minorities, HIV

## Abstract

Exposure to discrimination has been linked to lower HIV antiretroviral therapy (ART) adherence and poor HIV care outcomes among Black Americans. Coping has been shown to mitigate the harmful effects of discrimination on health behaviors, but the use of cultural relevant Africultural coping strategies is understudied as a moderator of the association between intersectional discrimination and ART adherence among Black Americans. We used adjusted logistic regression to test whether Africultural coping strategies (cognitive/emotional debriefing; collective; spiritual-centered; ritual-centered) moderated associations between multiple forms of discrimination (HIV, sexual orientation, race) and good ART adherence (minimum of 75% or 85% of prescribed doses taken, as measured by electronic monitoring in separate analyses) among 92 sexual minority Black Americans living with HIV. Mean adherence was 66.5% in month 8 after baseline (36% ≥ 85% adherence; 49% ≥ 75% adherence). Ritual-centered coping moderated the relationship between each of the three types of discrimination at baseline and good ART adherence in month 8 (regardless of the minimum threshold for good adherence); when use of ritual coping was low, the association between discrimination and adherence was statistically significant. The other three coping scales each moderated the association between racial discrimination and good ART adherence (defined by the 75% threshold); cognitive/emotional debriefing was also a moderator for both HIV- and race-related discrimination at the 85% adherence threshold. These findings support the benefits of Africultural coping, particularly ritual-centered coping, to help sexual minority Black Americans manage stressors associated with discrimination and to adhere well to ART.

**Supplementary Information:**

The online version contains supplementary material available at 10.1007/s10461-023-04233-7.

## Introduction

Disparities in HIV care outcomes among Black Americans include lower rates of antiretroviral therapy (ART) adherence [1]. Exposure to discrimination has been widely documented to impede health behavior [2], including ART adherence among Black Americans living with HIV [3–5]. The effect of discrimination on antiretroviral therapy (ART) adherence may be exacerbated among sexual minority Black Americans living with HIV, as they are exposed to multiple forms of discrimination and stigma— related to race, sexual orientation, and HIV status [3]—each of which has been associated with lower ART adherence [6].

Biopsychosocial models suggest that chronic discrimination affects health via detrimental responses to stress such as depression [7, 8] while adaptive coping strategies are posited to limit such responses to stress [9, 10]. Greater use of proactive coping (direct action; positive appraisal; support seeking) and less use of avoidant coping (behavioral disengagement; cognitive escape) have been found to help mitigate the harmful effects of discrimination on health outcomes among Black Americans [11–13]; however, several other studies have found null or contradictory results [2, 14].

Little research has examined the effects of culture-specific coping strategies commonly used by Black Americans to manage chronic stress from exposure to discrimination. African-centered coping strategies commonly used by Black Americans include spiritual practices and rituals (e.g., prayer, meditation, incense burning), support seeking from family and friends, and accessing culturally sanctioned social groups and community resources [15–17], which reflect West African coping principles such as spirituality, collectivism, and communalism [18, 19]. Findings have been mixed regarding whether the use of these strategies relate to levels of reported stress [20], and whether their use may serve as a buffer against the negative effects of discrimination on health outcomes [21, 22]. These studies used the Africultural Coping Systems Inventory (ACSI) [23], which measures coping strategies used to respond to discrimination specifically, and was developed and validated with mostly Black student populations; how well the scale operates with a clinical sample or persons living with a serious, chronic disease such as HIV is less established.

In the context of HIV, one study that used the Brief Religious Coping Scale [24] found that negative religious coping (i.e., attributing stressors to abandonment by or punishment from a higher power) was associated with greater psychological distress and lower ART adherence among Black Americans, while positive religious coping (i.e., collaborating with the higher power to navigate stressors) was associated with higher adherence [25]. We are not aware of any published studies of whether Africultural coping strategies can mitigate the harmful effects of discrimination on ART adherence.

With a convenience sample of sexual minority Black Americans living with HIV, and longitudinally monitored ART adherence, we examined multiple forms of discrimination (racial, HIV, sexual minority) as predictors of ART adherence, and whether the use of Africultural coping strategies (measured with the ACSI) moderates these associations. Understanding the role of Africultural coping strategies in managing discrimination will have implications for programs designed to help sexual minority Black Americans living with HIV to better cope with discrimination, and in turn better adhere to ART and manage their HIV disease.

## Methods

### Study Design

Participants selected for the analysis reported here were drawn from the participant pool of a randomized controlled trial of a community-based motivational interviewing intervention to improve ART adherence among Black Americans living with HIV. Participants were randomly assigned (using a 1:1 ratio) to either the intervention or usual care control arm. The multi-session, culturally tailored adherence counseling intervention was completed by month 6 and is described in greater detail in a prior publication [26]. The trial is registered with clinicaltrials.gov (NCT03331978).

Follow-up assessments occurred over 13 months, but only baseline assessment data (collected with Questionnaire Development Survey audio computer-assisted interview software) is used in this analysis, along with adherence data collected in the eight-month of follow-up. Incentives were provided in the form of gift cards to local businesses: $30 at baseline and $20 to $50 at each subsequent follow-up visit depending on the time point. The RAND Human Subjects Protection Committee approved the study.

### Study Setting and Participants

The study was conducted between January 2018 and December 2021 (with recruitment and enrollment from January 2018 to July 2020) at a large community-based HIV services organization in Los Angeles, AIDS Project Los Angeles Health & Wellness (APLA Health). As of December 31, 2021, Black individuals had the highest rates of new diagnoses, and the lowest ART adherence and viral suppression rates of all races/ethnicities in Los Angeles County [27]. Participants were recruited through outreach to program staff and clients (at the partner organization, other local organizations, and community events); in-reach (internal client referral in the partner organization); and social media outreach (e.g., on Facebook).

Eligibility criteria included: (1) Black/African American racial/ethnic identity, (2) HIV-positive serostatus, (3) 18 years of age or older, (4) prescribed ART for at least 6 months (so that baseline adherence would likely be stable), (5) self-reported adherence problems (i.e., missed at least one ART dose in the past month) and/or detectable viral load (based on biological assessment in the last 6 months); (6) willing to use an electronic adherence monitoring device; and (7) able to communicate in written and spoken English. For the analysis reported here, the criterion of self-identifying as a sexual minority individual (i.e., not heterosexual) was added to determine inclusion in the analytic dataset. Eligible participants were administered consent procedures by the study coordinator, and all enrolled participants provided written informed consent.

### Measures

#### ART Adherence

Daily adherence to one antiretroviral was monitored using a Medication Event Monitoring System (MEMS) bottle cap (AARDEX, Inc.) that records bottle opening dates and times. At baseline, participants received a MEMS cap to be used with one antiretroviral medication. For participants on more than one antiretroviral medication, the medication with the more complex dosing schedule, or the base medication if all medications had the same schedule, was used [28]. At each study visit, data from the cap were downloaded, and participants were asked to report how often the cap was not used as intended in the past two weeks (e.g., bottle opened without removing a dose or when multiple doses were removed at once), and responses were used to adjust the data at primary assessment time points [29]. Software associated with the cap calculates the proportion of prescribed doses taken during the specified time frame; for analysis, we used a dichotomous transformation of the data during the eighth month after baseline, operationalized as adherence to at least 75% of doses and 85% of doses, in separate variables. These cutoffs were chosen based on research suggesting virologic suppression is sustainable at these levels of adherence and that clinical benefit is received at higher adherence levels even though viral suppression can be sustained with lower adherence [30, 31].

#### Discrimination

The Multiple Discrimination Scale (MDS) [3] was used to assess experiences with ten different discrimination events in the past year due to race (MDS-Race), HIV-serostatus (MDS-HIV), and others’ perception of the respondent’s sexual orientation (MDS-Gay), with yes/no response options for each item. The events cover concrete behavioral expressions of prejudice, including interpersonal (from close others, partners, strangers, in general), institutional (verbal, employment, housing, healthcare), and violent (physical, property) forms of discrimination. The same set of 10 items were asked for each of MDS-Black/race (Cronbach’s alpha = 0.90), MDS-HIV (Cronbach’s alpha = 0.86), and MDS-gay (Cronbach’s alpha = 0.92). The sum of each subscale was computed; higher scores represented more discrimination events in the past year.

#### Africultural Coping

The 30-item Africultural Coping Systems Inventory (ACSI) [23] was used to measure four types of culture-specific coping: (1) cognitive and emotional debriefing (11 items; e.g., “Hoped that things would get better with time”; Cronbach’s alpha = 0.91); (2) collective coping (8 items; e.g., “Asked for suggestions on how to deal with the situation during a meeting of your organization or club”; Cronbach’s alpha = 0.89); (3) spiritual-centered coping (8 items; e.g., “Prayed that things would work themselves out”; Cronbach’s alpha = 0.90); and (4) ritual-centered coping (3 items; e.g., “Lit a candle for strength or guidance in dealing with the problem”; Cronbach’s alpha = 0.80). Participants were asked to indicate the extent to which they used each coping strategy (from 0 ‘did not use’ to 3 ‘used a great deal’) when faced with racism or discrimination due to their race, in the past year. If the respondent reported not experiencing any racial discrimination in the past year, they were instructed to select ‘not applicable’. For each subscale, a mean item score was calculated.

#### Socio-demographics

These included age, gender, education level (coded as less than high school graduate vs. high school graduate), annual income (coded as <$10,000 vs. $10,000 or greater), employment status (coded as full-time or part-time work vs. not working), housing status in last 12 months [coded as stable (e.g., own or rent) vs. unstable (e.g., homeless)], marital/relationship status (coded as married / cohabitating with partner vs. not), sexual orientation, and any incarceration as an adult (from age 18 years).

#### HIV Characteristics

Time since HIV diagnosis was self-reported. HIV viral load was assessed at baseline with blood drawn by the study coordinator, or via a copy of the participant’s latest laboratory results obtained from their primary care provider.

### Data Analysis

Nonresponse weights were developed to account for any potential bias introduced by restricting the baseline sample to participants who: (a) had responses to at least one of the four coping scales at baseline; and (b) provided electronic monitoring adherence data for the eighth month following baseline. These weights were derived as the inverse of predicted probabilities from a logistic regression on a variety of sociodemographic characteristics. All analyses other than descriptives and bivariate associations among baseline measures employed these nonresponse weights.

A series of weighted logistic regression models, with ART adherence in month 8 as the outcome, were run to assess associations with each of the three discrimination and four coping scales at baseline. Adherence during the eighth month of follow-up was used in the analysis, as this is the first month post completion of the intervention; this allowed us to control for the intervention effect on adherence as well as to examine the effects of discrimination over and above the immediate effect of the intervention. Further, the use of a MEMS caps to monitor adherence can artificially lead to increased adherence in the early weeks of its use [32]; therefore, using adherence during months further into study follow-up may result in a measurement of the participant’s more typical adherence patterns.

We examined whether each of the four coping scales moderated the association between each of the three types of discrimination and adherence. To do so, we conducted weighted logistic regression models with month 8 ART adherence as the outcome variable, and the baseline measures of the specific discrimination type and the specific coping scale, as well as the interaction of the discrimination and coping measures, with separate models conducted for each pairing of discrimination type and coping scale. Intervention condition was controlled for in each of these models, along with demographic covariates (age, gender, any college education), and all models used a complete-case approach. For models where the interaction term was significantly associated with good ART adherence at p < .05, post-estimation contrasts were performed on the sum of the effects for discrimination and the interaction with the coping moderator at its mean, one standard deviation above the mean, and one standard deviation below the mean to evaluate the effect of discrimination at low, medium, and high levels of the moderator. Two sets of analyses were conducted, one with the 75% cutoff for good adherence, and one with the 85% cutoff. Furthermore, a sensitivity analysis was conducted in which each of the above analyses was replicated, but with adherence during the thirteenth month after baseline. For all models, correction for multiple comparisons was conducted using the False Discovery Rate method [33].

Though not necessary for a moderation analysis, to better understand the relationship between discrimination and coping, we also ran a series of weighted linear regression models, with each baseline coping scale as the outcome in separate models, to assess associations with baseline HIV-, race- and sexual orientation-related discrimination; these models did not adjust for intervention condition, given that only baseline measures were in the models.

**Table 1:** Baseline sample characteristics among sexual minority participants (*N* = 92)

Characteristic	Mean (*SD*) or %
Age	49.4 (11.2)
Female	2.2%
Sexual orientation	
Gay or homosexual man or same gender loving	77.2%
Lesbian or homosexual woman	1.1%
Bisexual	21.7%
Educational attainment	
7th through 11th grade	9.8%
High school diploma or GED	21.7%
Some college, but no degree	39.1%
College degree	21.7%
Some graduate coursework, but no degree	5.4%
Graduate degree	2.2%
Employment status	
Employed, working 40 or more hours per week	5.4%
Employed, working 1–39 h per week	12.0%
Not employed, looking for work	18.5%
Not employed, not looking for work	5.4%
Retired	9.8%
Disabled, not able to work	43.4%
Student	5.4%
No unstable housing in the past year	55.4%
Annual household income	
I currently have no income	11.1%
More than $0 but less than $10,000	28.9%
$10,000-$20,000	46.7%
$20,001-$30,000	8.9%
$30,001-$40,000	2.2%
Over $40,000	2.2%
Years since HIV diagnosis	17.7 (10.0)
Marital status	
Single	83.5%
Living with Significant Other	4.4%
Married	1.1%
Divorced / Separated	11.0%
Widowed	0.0%
Ever been incarcerated since age 18	48.9%
Viral suppression	85.3%
Discrimination in the past year	
Due to race: experienced any events	51.1%
Due to race: number of events	2.0 (2.7)
Due to HIV status: experienced any events	58.2%
Due to HIV status: number of events	1.2 (2.1)
Due to orientation: experienced any events	69.2%
Due to orientation: number of events	1.3 (2.4)
Africultural coping (possible range 0–3)	
Cognitive / emotional	0.86 (0.75)
Collective	0.80 (0.76)
Ritual-centered	0.54 (0.76)
Spiritual-centered	0.87 (0.84)
ART adherence in month 8	
Took at least 75% of prescribed medications	48.9%
Percent of prescribed medications taken	66.5 (30.8)

SD = standard deviation

**Table 2:** Results from weighted linear regression modeling baseline measures of the Africultural coping strategies with baseline measures of HIV-, sexual-orientation-, and race-related discrimination

Type of coping	HIV Discrimination		Sexual Orientation Discrimination		Race Discrimination	
	Beta (SE)	p	Beta (SE)	p	Beta (SE)	p
Cognitive/emotional debriefing	**0.13 (0.03)**	**< 0.001**	**0.09 (0.03)**	**< 0.001**	**0.07 (0.03)**	**0.04**
Collective coping	**0.14 (0.03)**	**< 0.001**	**0.10 (0.03)**	**< 0.001**	**0.08 (0.03)**	**0.008**
Ritual-centered coping	**0.14 (0.05)**	**0.01**	**0.09 (0.04)**	**0.01**	0.05 (0.04)	0.20
Spiritual-centered coping	**0.16 (0.03)**	**< 0.001**	**0.12 (0.03)**	**< 0.001**	**0.08 (0.04)**	**0.03**

SE = standard error; bold type signifies statistical significance

## Results

### Sample Characteristics

Of the 565 individuals screened, 345 were eligible and 304 attended the first study visit in which they completed the baseline survey and received a MEMS cap for electronic adherence monitoring; 245 returned for the one-month visit and thus were randomized (122 intervention, 123 control). Of 245 randomized, 203 provided responses on at least one of the coping measures at baseline (based on the presence of discrimination events in the past year), of whom 147 self-identified as being a sexual minority individual (i.e., not heterosexual). Of these 147, electronic monitoring adherence data for month 8 was provided by 92 (47 intervention, 45 control) participants, and this subgroup comprised the analytic sample for this analysis.

Table 1 lists the baseline characteristics of the sample. Nearly all participants were male (98%), 55% were currently in a stable housing situation (e.g., rent or own their residence), and 49% had any history of incarceration. Mean years living with HIV was 17.7 (SD = 10.0), and 85% had an undetectable HIV viral load. As for the primary variables of interest in our analysis, the mean electronic monitored adherence level in month 8 was 66.5% (SD = 30.8%), with 49% and 36% achieving at least 75% and 85% adherence, respectively; half (51.1%) of the sample had experienced any race-related discrimination events in the past year (mean of 2.0 events), 58.2% had experienced any discrimination related to their HIV status (mean of 1.2 evens) and 69.2% had experienced any discrimination related to others’ perception of them being gay (mean of 1.3 events). The means of the four coping scales were all less than 1, suggesting that the coping strategies were generally used infrequently. None of these sample characteristics differed significantly between intervention and control arms (data not shown).

### Associations of Good ART Adherence (Defined Using Cutoffs of 75% and 85%) with Multiple Types of Discrimination and Africultural Coping

Logistic regression analysis, controlling for the effect of the intervention, revealed that good ART adherence (defined using 75% or greater prescribed doses taken) in month 8 was marginally associated with baseline discrimination related to HIV status [OR (95% CI) = 0.80 (0.63, 1.01); p = .06], but not significantly associated with discrimination related to either perceived gay sexual orientation [OR (95% CI) = 0.85 (0.69, 1.04); p = .12] or race [OR (95% CI) = 0.90 (0.77, 1.05); p = .19]. Logistic regression analysis also revealed that good ART adherence was not significantly associated with any of the baseline Africultural coping measures [cognitive / emotional coping, OR (95% CI) = 0.79 (0.44, 1.44); p = .44; collective coping, OR (95% CI) = 0.68 (0.38, 1.23); p = .20; ritual-centered coping, OR (95% CI) = 0.63 (0.31, 1.29); p = .20; spiritual-centered coping, OR (95% CI) = 0.81 (0.47, 1.40); p = .45]. Similarly, when 85% of prescribed doses taken was used to define good adherence, it was not found to be significantly associated with baseline measures of any of the types of discrimination nor any of the coping subscales (data not shown).

### Africultural Coping as a Moderator of the Association between Discrimination and Good ART Adherence (Defined as at Least 75% Doses Taken)

Linear regression analysis revealed that baseline measures of HIV-, race- and sexual orientation-related discrimination were each positively correlated with each of the four Africultural coping scales at baseline, with the exception of the nonsignificant association between ritual-centered coping and race-related discrimination (see Table 2). In logistic regression analysis to examine each coping scale as a moderator of the associations between good ART adherence (defined using the 75% cutoff) in month 8, and each discrimination type at baseline, only ritual-centered coping was a significant moderator for both HIV- and sexual orientation-related discrimination (see Tables 3 and 4), while all four coping scales were significant moderators for racial discrimination (see Table 5).

**Fig. 1:**
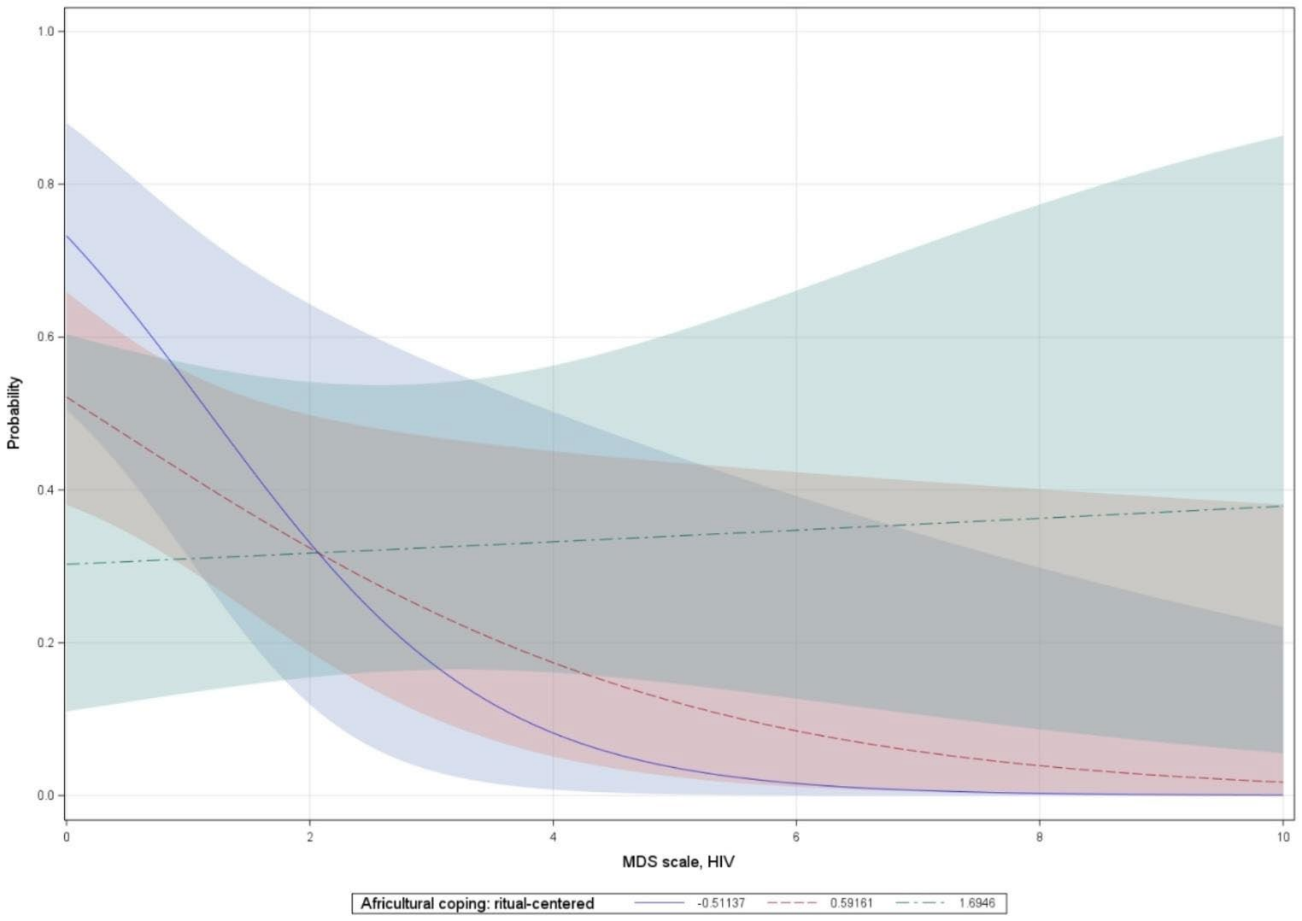
Predicted probabilities for at least 75% ART adherence in Month 8 (with 95% confidence intervals) based on baseline HIV discrimination

**Fig. 2:**
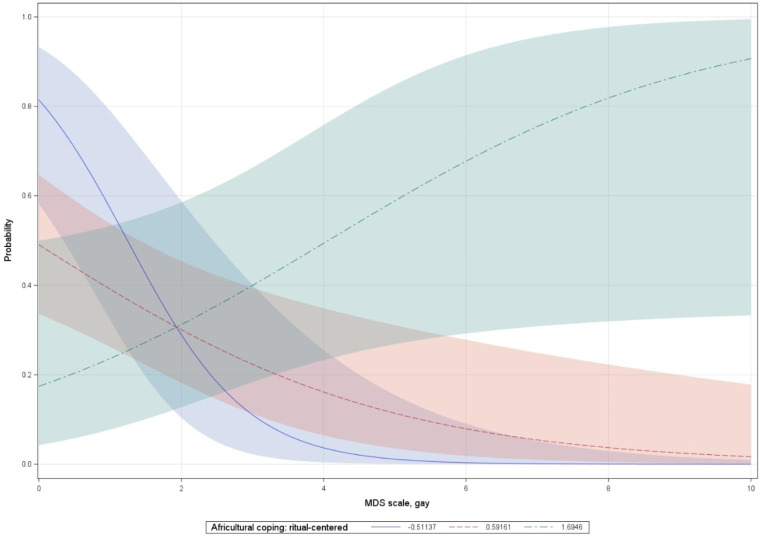
Predicted probabilities for at least 75% ART adherence in Month 8 (with 95% confidence intervals) based on baseline sexual orientation-related discrimination

**Table 3:** Logistic regression analysis^1^ of the interaction of baseline measures of HIV-related discrimination with each Africultural coping strategy as predictors of good ART adherence in month 8, by cutoff (75% and 85%) for minimum prescribed doses taken to define good adherence

	Good ART adherence defined as at least 75% prescribed doses taken				
		Effect of HIV-related discrimination on adherence when level of coping is…			
Type of coping	Interaction of discrimination and coping Log-odds (SE), p	Low (1 SD below the mean) OR (CI), p	Medium (mean) OR (CI), p	High (1 SD above the mean) OR (CI), p	
Cognitive/emotional debriefing	0.27 (0.21), p = .20				
Collective coping	0.31 (0.23), p = .17				
Ritual-centered coping	**0.40 (0.16), p = .02**	**0.42 (0.22–0.83), p = .01**	0.66 (0.45–0.97), p = .03	1.03 (0.76–1.41), p = .83	
Spiritual-centered coping	0.22 (0.21), p = .30				
	**Good ART adherence defined as at least 85% prescribed doses taken**				
		Effect of HIV-related discrimination on adherence when level of coping is…			
Type of coping	Interaction of discrimination and coping Log-odds (SE), p	Low (1 SD below the mean) OR (CI), p	Medium (mean) OR (CI), p	High (1 SD above the mean) OR (CI), p	
Cognitive/emotional debriefing	**0.59 (0.25), p = .02**	**0.25 (0.09–0.71), p = .01**	**0.47 (0.27–0.82), p = .009**	0.89 (0.64–1.23), p = .46	
Collective coping	0.44 (0.35), p = .21				
Ritual-centered coping	**0.59 (0.21), p = .005**	**0.27 (0.09–0.74), p = .01**	0.51 (0.27–0.97), p = .04	0.97 (0.63–1.50), p = .88	
Spiritual-centered coping	0.47 (0.30), p = .12				

^1^ Models included main effects of discrimination and coping, and were adjusted for intervention arm, age, gender, and education

SE = standard error, OR = odds ratio, CI = 95% confidence interval. Statistics are bolded if statistical significance remained after accounting for multiple comparisons with the False Discovery Rate method

**Table 4:** Logistic regression analysis^1^ of the interaction of baseline measures of sexual orientation-related discrimination with each Africultural coping strategy as predictors of good ART adherence in month 8, by cutoff (75% and 85%) for minimum prescribed doses taken to define good adherence

	Good ART adherence defined as at least 75% prescribed doses taken			
		Effect of sexual orientation-related discrimination on adherence when level of coping is…		
Type of coping	Interaction of discrimination and coping Log-odds (SE), p	Low (1 SD below the mean) OR (CI), p	Medium (mean) OR (CI), p	High (1 SD above the mean) OR (CI), p
Cognitive/emotional debriefing	0.32 (0.27) p = .24			
Collective coping	0.40 (0.23), p = .09			
Ritual-centered coping	**0.71 (0.20), p < .001**	**0.30 (0.16–0.57), p < .001**	**0.67 (0.51–0.88), p = .005**	1.47 (0.99–2.17), p = .06
Spiritual-centered coping	0.23 (0.20), p = .27			
	**Good ART adherence defined as at least 85% prescribed doses taken**			
		Effect of sexual orientation-related discrimination on adherence when level of coping is…		
Type of coping	Interaction of discrimination and coping Log-odds (SE), p	Low (1 SD below the mean) OR (CI), p	Medium (mean) OR (CI), p	High (1 SD above the mean) OR (CI), p
Cognitive/emotional debriefing	0.37 (0.34) p = .28			
Collective coping	0.54 (0.26), p = .04			
Ritual-centered coping	**0.65 (0.17), p < .001**	**0.33 (0.19–0.56), p < .001**	0.67 (0.48–0.94), p = .02	1.38 (0.87–2.18), p = .17
Spiritual-centered coping	0.27 (0.29), p = .36			

^1^ Models included main effects of discrimination and coping, and were adjusted for intervention arm, age, gender, and education

SE = standard error, OR = odds ratio, CI = 95% confidence interval. Statistics are bolded if statistical significance remained after accounting for multiple comparisons with the False Discovery Rate method

**Table 5:** Logistic regression analysis^1^ of the interaction of baseline measures of race-related discrimination with each Africultural coping strategy as predictors of good ART adherence in month 8, by cutoff (75% and 85%) for minimum prescribed doses taken to define good adherence

	Good ART adherence defined as at least 75% prescribed doses taken				
		Effect of race-related discrimination on adherence when level of coping is…			
Type of coping	Interaction of discrimination and coping Log-odds (SE), p	Low (1 SD below the mean) OR (CI), p	Medium (mean) OR (CI), p	High (1 SD above the mean) OR (CI), p	
Cognitive/emotional debriefing	**0.32 (0.12), p = .01**	**0.60 (0.41–0.88), p = .009**	0.85 (0.70–1.03), p = .10	1.21 (0.92–1.57), p = .17	
Collective coping	**0.37 (0.14), p = .01**	**0.55 (0.36–0.84), p = .007**	0.83 (0.68–1.01), p = .06	1.24 (0.92–1.66), p = .15	
Ritual-centered coping	**0.41 (0.15), p = .007**	**0.58 (0.39–0.86), p = .008**	0.92 (0.74–1.13), p = .41	1.45 (0.98–2.13), p = .06	
Spiritual-centered coping	**0.27 (0.11), p = .02**	**0.61 (0.42–0.90), p = .01**	0.84 (0.69–1.02), p = .08	1.16 (0.88–1.52), p = .29	
	**Good ART adherence defined as at least 85% prescribed doses taken**				
		Effect of race-related discrimination on adherence when level of coping is…			
Type of coping	Interaction of discrimination and coping Log-odds (SE), p	Low (1 SD below the mean) OR (CI), p	Medium (mean) OR (CI), p	High (1 SD above the mean) OR (CI), p	
Cognitive/emotional debriefing	**0.32 (0.14), p = .02**	**0.60 (0.38–0.93), p = .02**	0.84 (0.68–1.04), p = .11	1.18 (0.90–1.55), p = .22	
Collective coping	0.32 (0.16), p = .04				
Ritual-centered coping	**0.59 (0.14), p < .001**	**0.50 (0.34–0.75), p < .001**	0.96 (0.77–1.19), p = .71	**1.84 (1.30–2.59), p < .001**	
Spiritual-centered coping	0.21 (0.13), p = .11				

^1^ Models included main effects of discrimination and coping, and were adjusted for intervention arm, age, gender, and education

SE = standard error, OR = odds ratio, CI = 95% confidence interval. Statistics are bolded if statistical significance remained after accounting for multiple comparisons with the False Discovery Rate method

**Fig. 3:**
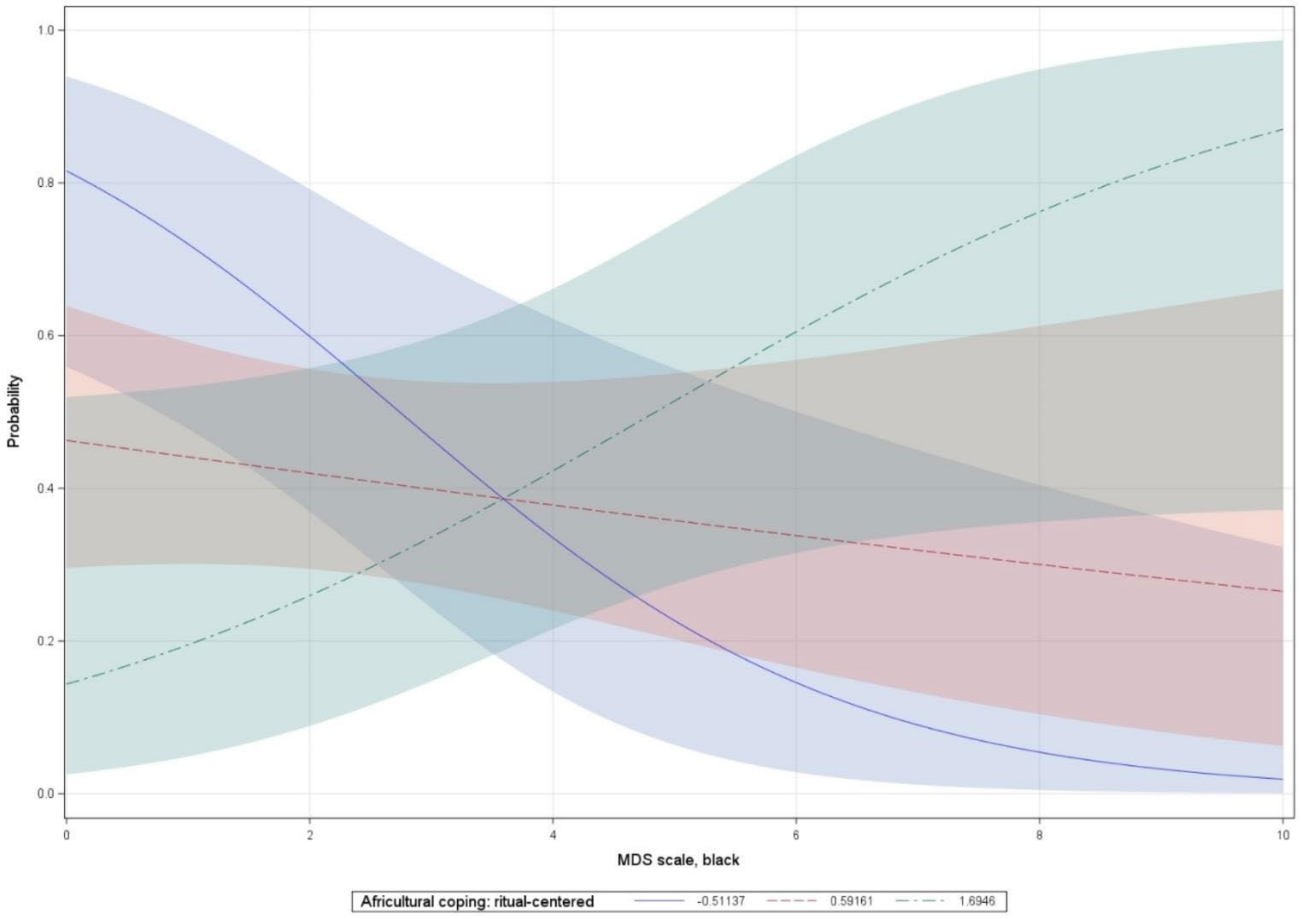
Predicted probabilities for at least 75% ART adherence in Month 8 (with 95% confidence intervals) based on baseline racial discrimination

As shown in Tables 3, 4 and 5 and depicted in Figs. 1, 2 and 3, regression analyses that estimated the effect of each type of discrimination at low, medium and high levels of ritual-centered coping, revealed that each type of discrimination had a weaker negative effect on adherence as the use of ritual-centered coping increased, with a consistent significant effect when ritual-centered coping was at a low level. Similarly, analysis of the effect of racial discrimination at low, medium and high levels of collective coping, cognitive/emotional debriefing, and spiritual-centered coping, showed that discrimination had a weaker negative effect on adherence as the use of each coping style increased, but the effect was only significant when the use of the coping style was at a low level (the graphs of these patterns can be found online in the supplementary materials).

### Africultural Coping as a Moderator of the Association between Discrimination and Good ART Adherence (Defined as at Least 85% Doses Taken)

When the moderation analysis was repeated with good adherence defined as at least 85% of prescribed doses taken, ritual-centered coping was a significant moderator of the relationship between good adherence in month 8 and each type of discrimination. Each type of discrimination had a weaker negative effect on adherence as the use of ritual-centered coping increased, with a significant effect when ritual-centered coping was at a low level (see Tables 3, 4 and 5); a significant effect at a high level of ritual-centered coping was also found in the analysis of racial discrimination.

Cognitive/emotional debriefing was also a significant moderator of the relationship between good adherence and both HIV- and race-related discrimination. HIV- and race-related discrimination each had a weaker negative effect on adherence as the use of cognitive/emotional debriefing increased, with the effect of HIV-related discrimination being significant at low and moderate levels of the coping style, while the effect of racial discrimination was only significant when the use of the coping style was at a low level (see Table 5).

When this moderation analysis was replicated with adherence data in the thirteenth month (with a sample of 69 sexual minority participants who provided data in this month), none of the coping scales moderated the association between any of the three types of discrimination and ART adherence (regardless of whether 75% or 85% cutoff was used). The data are available in online supplementary materials.

## Discussion

In this sample of mostly male, sexual minority Black Americans living with HIV who generally had low ART adherence, the use of multiple types of Africultural coping strategies was examined as a moderator of the relationship between three types of discrimination (HIV, race, perceived gay sexual orientation) and good ART adherence. Each type of discrimination was uncorrelated with good adherence when coping was not accounted for; however, when the use of Africultural coping strategies was low, each form of discrimination emerged as a significant predictor of adherence. This was particularly true for ritual-centered coping strategies, which was the only coping method that moderated the association between each type of discrimination and good ART adherence (regardless of whether good adherence was defined using a cutoff of at least 75% or 85% of prescribed doses taken), suggesting that this form of coping helps to mitigate the harmful effects of these forms of discrimination on adherence.

Several studies have found that proactive coping (e.g., active problem solving) helps to mitigate the harmful effects of discrimination on health outcomes among Black Americans [12, 14]. African-centered forms of coping, such as participation in prayer and meditation, seeking support from family and friends, and connecting with culturally sanctioned social groups and community resources, are commonly used by Black Americans to cope with stress [15–17], including those living with HIV [25, 43]. However, this study may be the first to examine whether Africultural coping methods mitigate the harmful effect of discrimination on ART adherence, thereby acting as a moderator of the association between discrimination and adherence. Our data showed that ritual-centered coping consistently moderated the associations between HIV-, race- and sexual orientation-related discrimination and ART adherence, with each type of discrimination having a negative effect on adherence with lower use of ritual-centered coping (e.g., burning incense for strength, or using a cross or other object for its special powers), but not when this coping was at a high level. Ritual-based coping has been found to be associated with reduced stress in other studies, though not in the context of HIV [44].

The other coping scales (cognitive/emotional debriefing, collective coping, spiritual-centered coping) each acted as moderators of the association between racial discrimination and good ART adherence when good adherence was defined as at least 75% of prescribed doses taken. Cognitive/emotional debriefing was also a moderator for both HIV- and race-related discrimination at the higher threshold (85% cutoff) for good adherence. These findings suggest that for racial discrimination in particular, these other forms of Africultural coping have a role to play in helping Black sexual minority Americans cope with discrimination and manage their HIV disease. Future research may benefit from including measures of mental health (e.g., depression, stigma, substance use) and resilience (e.g., social support), which have been associated with discrimination and may help further improve our understanding of how Africultural coping relates to various forms of discrimination and ART adherence [37–39].

It is important to note that each of the three forms of discrimination, while being widely experienced by the study participants, was not associated with good ART adherence when coping was unaccounted for, unlike what other research has found [6]. Each form of discrimination as a predictor of poor ART adherence emerged only in the context of low use of Africultural coping. Exposure to each of these forms of discrimination has been found to be associated with internalized stigma [34] and heightened stressors and mental health challenges (e.g., depression, substance use) [37–39], which can impede ART adherence and HIV disease management [35, 36, 40–42]. Our findings suggest that Africultural coping strategies may help serve as an important buffer against the harmful impact of these psychosocial factors, as the effects of these factors rise to prominence when the use of these coping strategies is low. Counseling and adherence interventions designed for Black sexual minority individuals living with HIV may benefit from including instruction on how to effectively use Africultural coping strategies.

The present analysis is one of the few studies to use the Africultural Coping Systems Inventory (ACSI; [23]) to measure coping in a sample of sexual minority individuals living with HIV. Internal reliability was strong for each coping subscale, and construct validity was supported by the significant associations between the coping subscales and the various forms of discrimination measured in this study, as well as with ART adherence. These findings suggest that the scale can be effectively used to examine coping among sexual minority individuals living with HIV. There are limitations to using the ACSI for assessing coping responses to multiple types of discrimination, which should be noted. The scale assesses coping strategies used in response to events of racial discrimination, yet our analysis examined correlations of the coping scales to other forms of discrimination as well (i.e., HIV- and sexual orientation-related discrimination). Moreover, we tested the effects of Africultural coping as a moderator, i.e., a pre-existing resilience resource that buffers the effects of stressful discrimination events, rather than a mediator that is a direct response to the stressor of discrimination, as some other research has done [21, 22]. Research has shown that coping strategies can differ across these distinct types of discrimination [45, 46]; however, the use of some forms of coping (e.g., seeking social support from family and friends) may be more generalizable across identities. Also, well-used strategies that have been refined across numerous discrimination experiences (e.g., a lifetime of racial discrimination), may serve as a resilience resource across types of discrimination. With most of the study sample exposed to discrimination related to their race, sexual orientation, and HIV status, discrimination must be viewed in an intersectional context, in which the person experiences discrimination holistically, across their various identities. From this perspective, individuals may not always be able to discern which identity the discrimination is attributed to, resulting in difficulty assessing whether coping strategies are applied to specific forms of discrimination [47]. Future research could adapt the ACSI to assess the use of Africultural coping to each specific form of discrimination being studied, or alternatively measure coping strategies to discrimination in general.

Other study limitations include our use of electronic monitoring caps to measure medication adherence; this methodology is an objective measure that has been found to be superior to self-reported ART adherence, but it may underestimate actual pill ingestion [48]. Also, our small convenience-based sample is limited in terms of statistical power and generalizability to the larger population of sexual minority Black Americans living with HIV.

In conclusion, in this sample of sexual minority Black Americans with generally poor ART adherence, the association between discrimination related to HIV status, race and sexual orientation and good ART adherence was only evident at low levels of use of Africultural coping strategies. Although each type of Africultural coping was found to moderate the relationship between at least one form of discrimination and good ART adherence, ritual-centered coping was the only coping method that was a significant moderator of the association between each form of discrimination and good ART adherence. This was true regardless of whether good adherence was defined by the minimal threshold of 75% or 85% prescribed doses taken. These findings provide evidence for the benefits of Africultural coping, and in particular ritual-centered coping, to help sexual minority Black Americans living with HIV to manage stressors associated with discrimination and to adhere well to ART. Further research is needed to better understand how ritual-centered coping has its buffering effect in protection against discrimination, and how ART adherence support programs for sexual minority Black Americans can best incorporate this form of coping as a response to discrimination.

### Electronic Supplementary Material

Below is the link to the electronic supplementary material.

Supplementary Material 1

Supplementary Material 2

## Data Availability

All authors ensure that the data support the published claims and comply with field standards.
