# Supplementary material for: Culturally Relevant Africultural Coping Moderates the Association Between Discrimination and Antiretroviral Adherence Among Sexual Minority Black Americans Living with HIV
Source: AIDS Behav. 2023 Dec 7;28(2):408–20. doi: 10.1007/s10461-023-04233-7 (PMC10876751; doi:10.1007/s10461-023-04233-7)

**Supplement Graphs**

**Figure S1.** Predicted probabilities for at least 75% ART adherence in Month 8 (with 95% confidence intervals) based on MDS scale (Black/Race) for Africultural coping (cognitive/emotional debriefing).


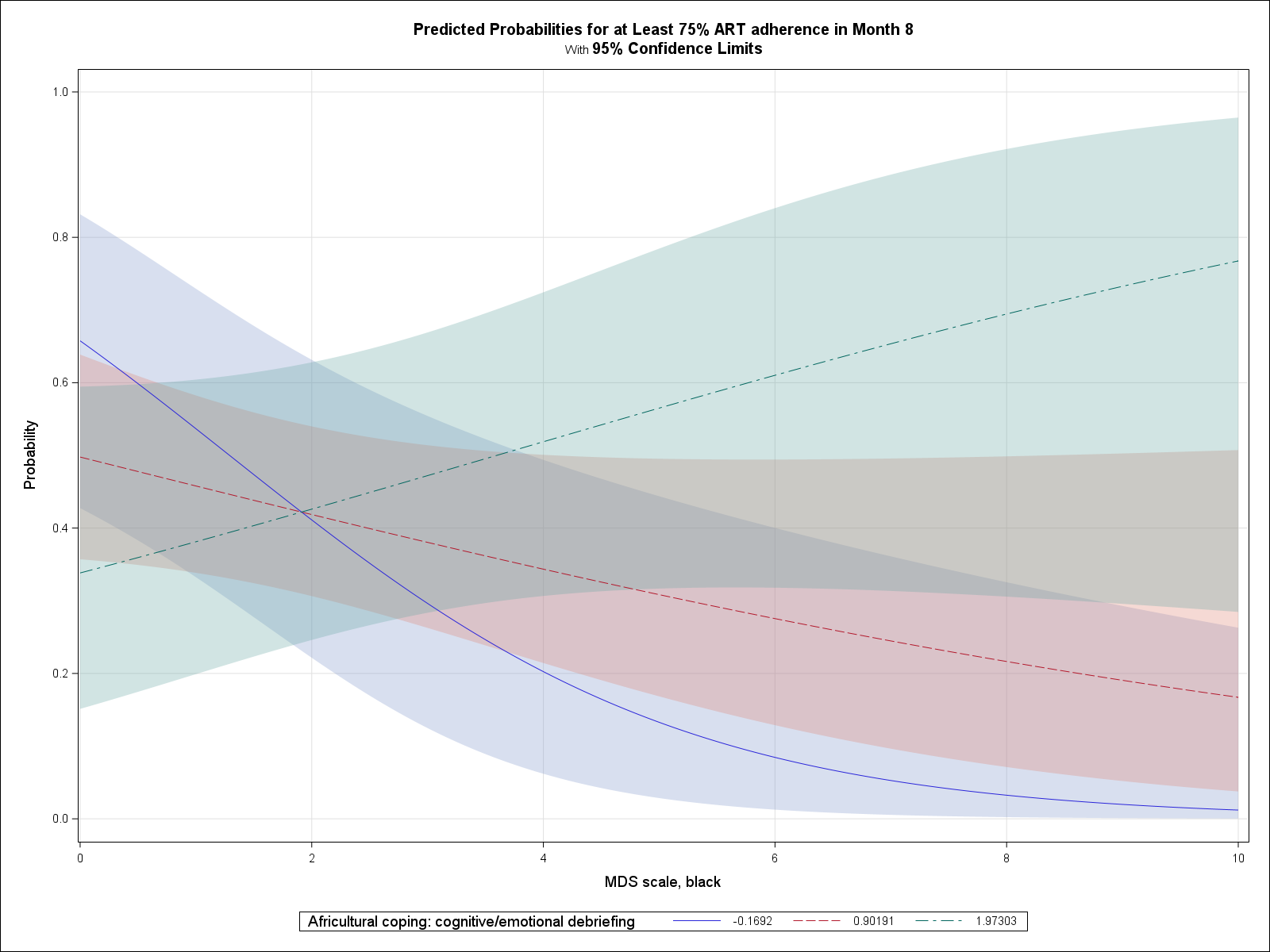


**Figure S1.** Predicted probabilities for at least 75% ART adherence in Month 8 (with 95% confidence intervals) based on MDS scale (Black/Race) for Africultural coping (collective).


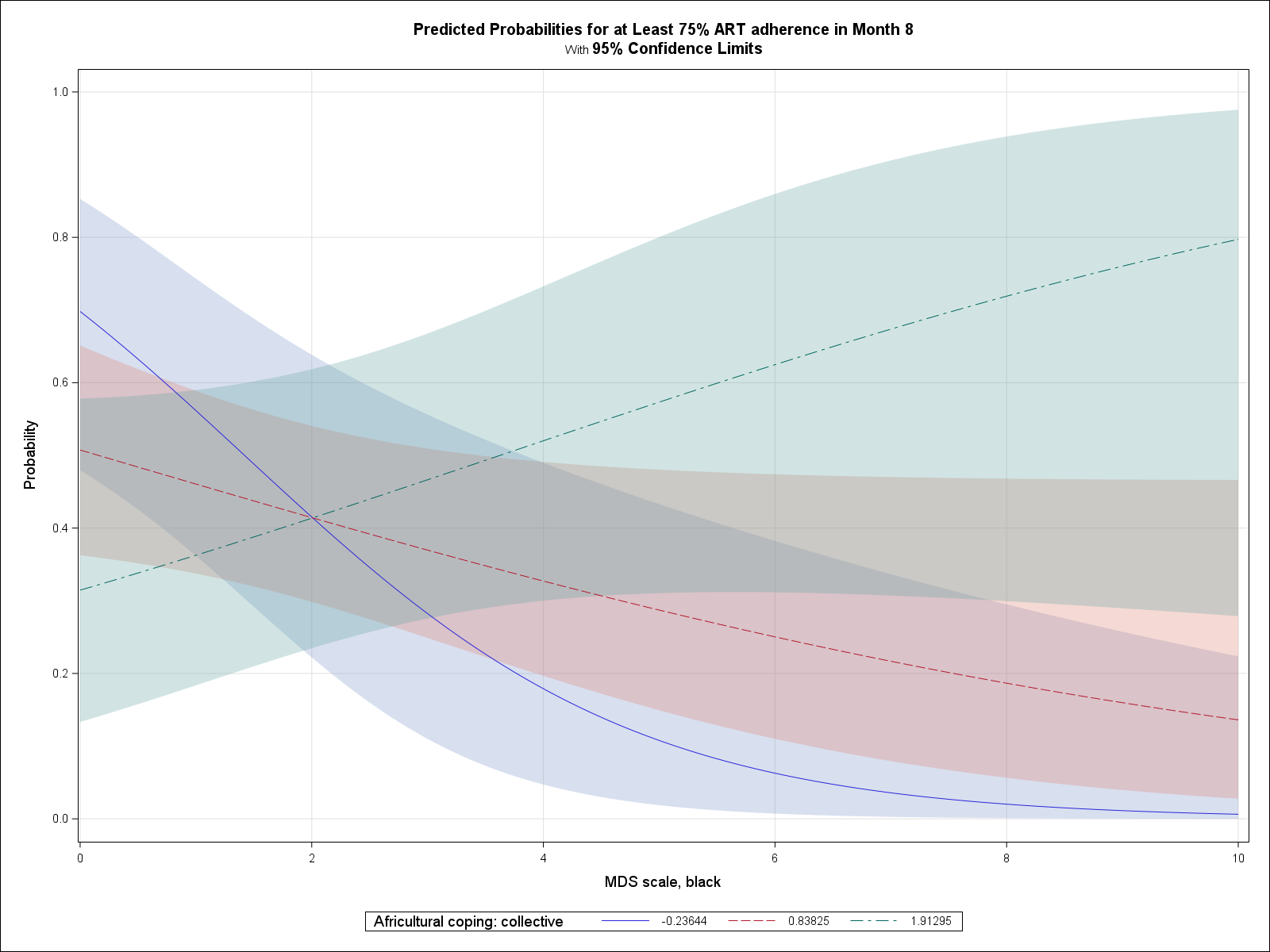


**Figure S3.** Predicted probabilities for at least 75% ART adherence in Month 8 (with 95% confidence intervals) based on MDS scale (Black/Race) for Africultural coping (spiritual-centered).


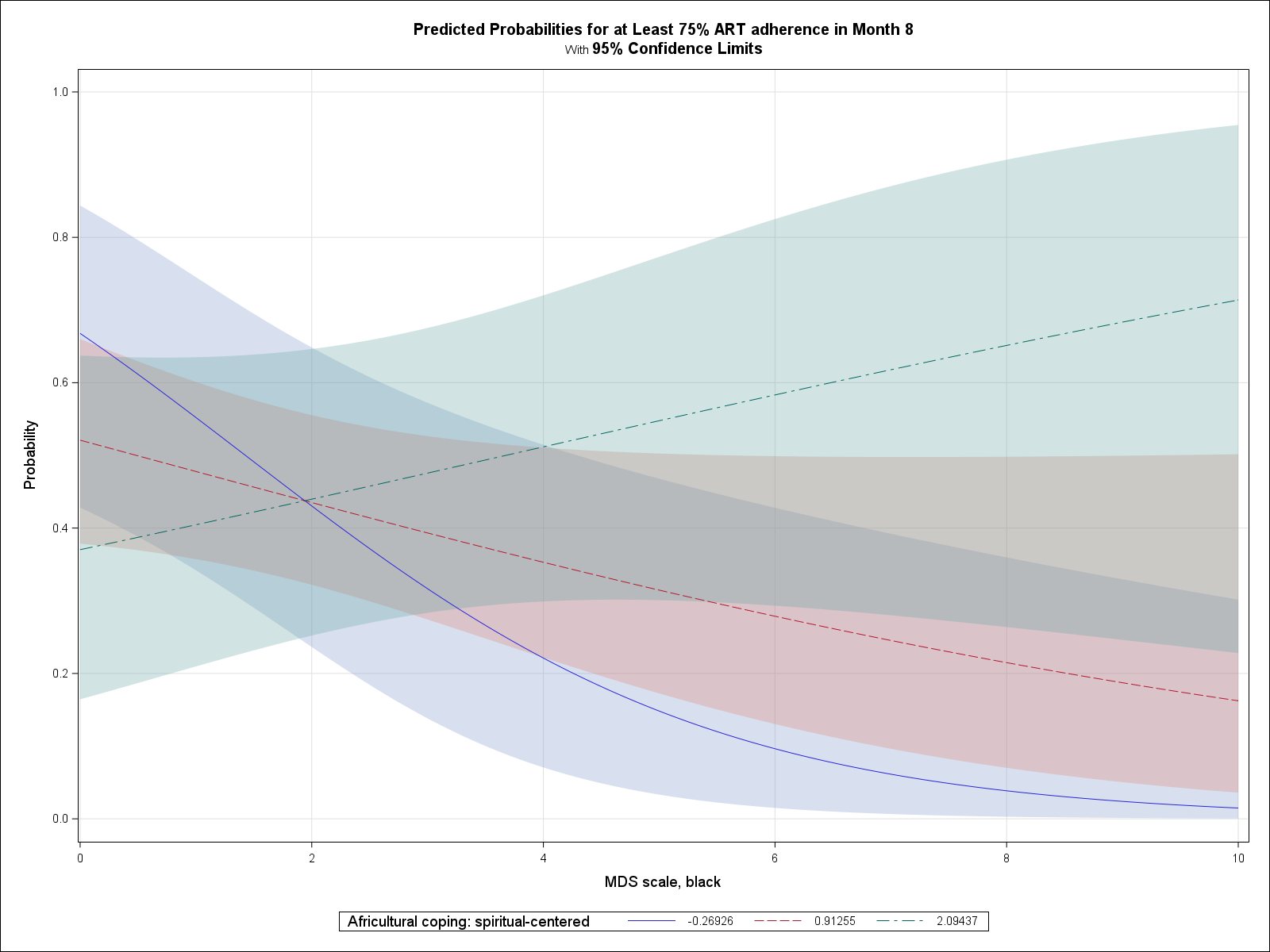

Supplement: Supplementary file 1 — Supplementary Material 1 [file 10461_2023_4233_MOESM1_ESM.docx]
