# Supplementary material for: Culturally Relevant Africultural Coping Moderates the Association Between Discrimination and Antiretroviral Adherence Among Sexual Minority Black Americans Living with HIV
Source: AIDS Behav. 2023 Dec 7;28(2):408–20. doi: 10.1007/s10461-023-04233-7 (PMC10876751; doi:10.1007/s10461-023-04233-7)
Supplement: Supplementary file 2 — Supplementary Material 2 [file 10461_2023_4233_MOESM2_ESM.docx]

**Table 3A. Logistic regression analysis^1^ of the interaction of baseline measures of HIV-related discrimination with each Africultural coping strategy as predictors of good ART adherence in month 13, by cutoff (75% and 85%) for minimum prescribed doses taken to define good adherence**

|  | Good adherence defined as > 75% | Good adherence defined as > 85% |
| --- | --- | --- |
| Type of coping | Interaction of discrimination and coping  Log-odds (SE), p | Interaction of discrimination and coping  Log-odds (SE), p |
| Cognitive/emotional debriefing | 0.02 (0.16), p=.92 | 0.00 (0.37), p=.99 |
| Collective coping | 0.13 (0.16), p=.43 | 0.43 (0.28), p=.13 |
| Ritual-centered coping | -0.41 (0.29), p=.16 | -0.07 (0.33), p=.84 |
| Spiritual-centered coping | 0.22 (0.19), p=.27 | 0.56 (0.42), p=.19 |

^1^ Models included main effects of discrimination and coping, and were adjusted for intervention arm, age, sex, and education.

SE = standard error, OR = odds ratio, CI = 95% confidence interval.

**Table 4B. Logistic regression analysis^1^ of the interaction of baseline measures of sexual orientation-related discrimination with each Africultural coping strategy as predictors of good ART adherence in month 13, by cutoff (75% and 85%) for minimum prescribed doses taken to define good adherence**

|  | Good adherence defined as > 75% | Good adherence defined as > 85% |
| --- | --- | --- |
| Type of coping | Interaction of discrimination and coping  Log-odds (SE), p | Interaction of discrimination and coping  Log-odds (SE), p |
| Cognitive/emotional debriefing | -0.19 (0.19) p=.31 | -0.84 (0.41) p=.046^2^ |
| Collective coping | 0.00 (0.13), p=.99 | 0.01 (0.18), p=.95 |
| Ritual-centered coping | -0.12 (0.18), p=.52 | 0.04 (0.14), p=.78 |
| Spiritual-centered coping | 0.02 (0.14), p=.86 | -0.12 (0.22), p=.59 |

^1^ Models included main effects of discrimination and coping, and were adjusted for intervention arm, age, sex, and education.

^2^ Statistical significance did not remain after adjusting for multiple comparisons using False Discovery Rate method.

SE = standard error, OR = odds ratio, CI = 95% confidence interval.

**Table 5B. Logistic regression analysis^1^ of the interaction of baseline measures of race-related discrimination with each Africultural coping strategy as predictors of good ART adherence in month 13, by cutoff (75% and 85%) for minimum prescribed doses taken to define good adherence**

|  | Good adherence defined as > 75% | Good adherence defined as > 85% |
| --- | --- | --- |
| Type of coping | Interaction of discrimination and coping  Log-odds (SE), p | Interaction of discrimination and coping  Log-odds (SE), p |
| Cognitive/emotional debriefing | -0.14 (0.15) p=.35 | -0.31 (0.22) p=.16 |
| Collective coping | -0.14 (0.15), p=.33 | -0.45 (0.20), p=.03^2^ |
| Ritual-centered coping | -0.22 (0.14), p=.12 | -0.16 (0.13), p=.23 |
| Spiritual-centered coping | -0.19 (0.14), p=.19 | -0.37 (0.18), p=.04^2^ |

^1^ Models included main effects of discrimination and coping, and were adjusted for intervention arm, age, sex, and education.

^2^ Statistical significance did not remain after adjusting for multiple comparisons using False Discovery Rate method.

SE = standard error, OR = odds ratio, CI = 95% confidence interval.
